# Algal production of extra and intra-cellular polysaccharides as an adaptive response to the toxin crude extract of *Microcystis aeruginosa*

**DOI:** 10.1186/1735-2746-9-10

**Published:** 2012-11-20

**Authors:** Mostafa Mohamed El-Sheekh, Hanan Mohamed Khairy, Rania El-Shenody

**Affiliations:** 1 Botany Department, Faculty of Science, Tanta University, Tanta, Egypt; 2 National Institute of Oceanography and Fisheries, Alexandria, Egypt

**Keywords:** Allelopathy, *Microcystis aeruginosa*, Crude extracts, Intra and extra- cellular polysaccharides, Cyanotoxins

## Abstract

This is an investigation concerned with studying the possible adaptive response of four different unicellular algae, *Anabaena* PCC 7120, *Oscillatoria angustissima*, *Scendesmus obliquus and Chlorella vulgaris*, to the toxin of *Microcystis aeruginosa* (Kützing). The effects of four different concentrations, 25, 50, 100 and 200 μg mL^-1^ of microcystins crude extract of *M*. *aeruginosa*, on both intra and extra-cellular polysaccharide levels, in log phase, of the four tested algae were studied. The obtained results showed differential increase in the production levels for both intra and extra-cellular polysaccharides by the tested algae, compared with the control. *S*. *obliquus* and *C*. *vulgaris* showed a resistance to crude toxin higher than *Anabaena* PCC 7120 and *O*. *angustissima*. The highly production of polysaccharides by green algal species under this toxic stress indicated the involvement of these polysaccharides in protecting the algal cells against toxic species and, reflect the biological behavior of particular algal species to the environmental stresses.

## Introduction

*Microcystis aeruginosa* is a common hepatotoxic cyanobacterium living in eutrophic freshwaters [1]. The inhibition of competitors by the release of compounds, a process known as allelopathy, may be important in plank tonic systems [2]. Allelopathy has been hypothesized to play a role in species succession [3], the formation of harmful algal blooms in water resources [4], and the establishment of invasive species [5]. Allelopathy is defined as any process involving secondary metabolites produced by plants and microorganisms that influence the growth and development of biological systems, including positive and negative effects [6]. These secondary metabolites are called allelochemicals and play a major role on growth and development in both natural and agro-ecosystems [7]. Most allelopathic are compounds biodegradable and at the same time natural toxins [8].

The production of biologically active substances which promotes the growth of algae and other plant organisms has been reported [9].

The algal cells can release extra-cellular polysaccharides (EPS) into the environment; these EPS are ecologically important through their influence on carbon cycle and microbial diversity [10]. They may enhance the bacterial growth and activity leading to the release of inorganic substances useful for microalgae in the same environment [11]. In addition, these polysaccharides can also make complexes with inorganic ions and thus reducing their toxicity to aquatic organisms [12].

Some green algae can develop a defense system by the production of polysaccharides to cope with oxidative stress induced by microcystin. The results of *in vitro* assay of antioxidant activity revealed that these polysaccharides had different activities, depending on their sulfate contents [13]. On the other hand, toxic blooms of *Nodularia spumigena* can provide a potential food source for the heterotrophic food chain [14]. Furthermore, bacteria can efficiently degrade microcystins in natural waters with previous cyanobacterial history, and heterotrophic nanoflagellates respond quickly to the bacterial growth [15]. However, some species of green algae e.g. *Scenedesmus* sp. coexist and even flourish in the presence of either toxic cyanobacteria or their toxins [16]. Mass-occurrences of cyanobacteria have been reported to increase in frequency, as well as in intensity, due to eutrophication [17]. It is therefore important to investigate how cyanobacteria influence on the structure and functioning of other surrounding organisms in the aquatic ecosystem since River water quality zoning could provide essential information for developing river water quality management policies [18].

Therefore, the aim of this study was to investigate the response of several unicellular algae to the stress of crude toxin of *Microcystis aeruginosa* through the production of intra and extra-cellular polysaccharides.

## Materials and methods

### Test organisms

Two unicellular blue green algae (*Anabaena* PCC. 7120 and *Oscillatoria angustissima*) and two green algae (*Scendesmus obliquus* and *Chlorella vulgaris*) were kindly provided from Phycology Laboratory, Faculty of Science, Tanta University.

### Culture and crude extract of cyanobacterium *Microcystis aeruginosa*

*Microcystis aeruginosa* was isolated from fresh water samples from Nile river channel near Tanta city, Egypt, and spread with an inoculating needle on the surface of sterilized Petri dishes containing solidified, sterile media (Allen’s, [19]). The cultures of *M*. *aeruginosa* purified and prepared in axenic unialgal cultures by Venkataraman [20].

*M*. *aeruginosa* was cultured in 1 liter conical flasks containing 400 mL medium (Allen’s, [19]) and kept in controlled conditions of continuous light (45 μmol/ms) and temperature (25±2°C). Algal cells were harvested at the end of log growth phase, lyophilized and kept in deep freeze (−20°C).

The crude extracts were prepared by suspending 100 mg lyophilized cells in 10 mL of 75% methanol according to [21] with ultrasonication (ultrasonic probe with characteristics of ~60 W and ~20 KHZ) for 2 min followed by intermittent shaking for 1 h. Debris was removed by centrifugation for 10 min at 4000 rpm. The pellet was reextracted and the combined supernatants were evaporated to dryness at 30°C using a rotary evaporator (Perfit, India).

### Treatments

All experiments were carried out in 250 mL conical flasks, containing 100 mL Allen’s and Stanier medium which was adjusted to contain 0 (control), 25, 50, 100 and 200 μg/mL of lyophilized cell extract of previously mentioned cyanobacteria species. Culture conditions of the tested algae were the same as previously mentioned. The experimental cultures were harvested at 3, 6, 9, 12 and 15 days for the determination of intra and extra-cellular polysaccharides. Cultures were grown in triplicate for statistical analysis.

### Measurement of intra-cellular polysaccharides (IPS)

During the growth, 100 mL of the tested algae cultures were pipette out and centrifuged at 3000 rpm for 10 min. The filtrate was used to estimate extra-cellular polysaccharides and the pellets were dried and then used to estimate intra-cellular polysaccharide as described by [22]. Intra-cellular polysaccharide (IPS) was extracted by homogenizing the derided pellet in distilled water (50 mL). The homogenates were then heated in water bath at 95°C for 6 hours. The extracts were filtrated through Whatman No.2 filter paper, then precipitated with four volumes of 95% ethanol, stirred vigorously and left overnight at 4°C. The precipitated IPS was recovered by centrifugation at 10.000 rpm for 15 min and the supernatant was discarded.

### Measurement of extra-cellular polysaccharide (EPS)

The extra-cellular polysaccharides were estimated according to [23]. To precipitate proteins from the algal culture, trichloroacetic acid (TCA) was added in a final concentration of 4% and the algal filtrate was stirred for 2 h. Precipitated proteins were removed by centrifugation. The clear supernatant was collected which contains EPS. Extra-cellular polysaccharides was precipitated by ethanol and determined as described in IPS.

### Statistical analysis

Results are presented as mean ±SD (standard deviation) for three replicates. Data obtained were analyzed statistically to determine the degree of significance between treatments using one, two and three way analysis of variance (ANOVA) at P ≤ 0.001. The statistical analyses were carried out using SAS program [24] version 6.12.

## Results

Data in Table 1 show the effect of various concentrations of microcystins crude extract of *M*. *aeruginosa* (25, 50, 100 and 200 μg/mL), in log phase, on both extra-cellular polysaccharides (EPS) and intra-cellular polysaccharides (IPS) of *Anabaena* PCC.7120. Results showed a significant increase of both EPS and IPS as compared with control. Thus, the crude microcystins extract could induce the production of polysaccharides and their amounts increased with increasing the microcystins crude extract concentration. At the end of the experiment (day15), the increase in EPS and IPS for *Anabaena* PCC.7120 amounted by (20%, 52.6%, 81.0% and 132.8%) and (17%, 57%, 91% and 112.6%) over control level for EPS and IPS at different concentrations, respectively. One way analysis of variance revealed a high significant increase in extra and intra-cellular polysaccharides of *Anabaena* PCC. 7120 as compared with control at (P ≤ 0. 001). On the other hand, in *Oscillatoria angustissima*, EPS and IPS increased, and amounted by 20%, 37%, 53% and 86.7% and 8.7%, 37.8%, 57.6% and 96%, respectively (Table 2). High significant increase in extra and intra-cellular polysaccharides at (P ≤ 0. 001) compared with the control at all concentrations of the microcystins crude extract except the first concentration 25 μg/mL, the increase in IPS at (all days of experiments) and EPS at 6 days were insignificant.

**Table 1:** **Effect of different concentrations of microcystins crude extract (25, 50, 100 and 200 μg/mL) of *Microcystis aeruginosa *** **in log phase on intra (IPS) and extra-cellular polysaccharides content (EPS) in μg/mL of *Anabaena *** **PCC. 7120**

**Microcystin crude extract concentrations (μg/mL)**								
**Polysaccharide**	**Days**	**Control**	**25**	**50**	**100**	**200**	**F value**	**P value**
	0	105±18	105±18	105±18	105±18	105±18		
	3	182±19	182±19^(ns)^	182±19^(ns)^	182±19^(ns)^	182±19^(ns)^		
IPS	6	275±22	393±34^***^	453±32^***^	529±28^***^	577±23^***^	1062.1	0.001
	9	523±50	620±23^***^	858±52^***^	1009±40^***^	1153±50^***^		
	12	767±34	948±26^***^	1239±39^***^	1533±35^***^	1667±40^***^		
	15	1087±78	1307±40^***^	1659±36^***^	1968±42^***^	2531±36^***^		
	0	81±20	81±20^(ns)^	81±20^(ns)^	81±20^(ns)^	81±20^(ns)^		
	3	150±21	150±21^(ns)^	150±21^(ns)^	150±21^(ns)^	150±21^(ns)^		
EPS	6	234±27	329±21^***^	427±24^***^	473±37^***^	543±27^***^	255.86	0.001
	9	334±31	426±28^***^	516±41^***^	618±48^***^	716±24^***^		
	12	397±25	481±19^***^	585±44^***^	702±40^***^	801±25^***^		
	15	476±58	557±27^***^	748±37^***^	909±25^***^	1012±45^***^		

^***^ Highly significant at P ≤ 0.001 using one way analysis of variance (ANOVA).

^(ns)^ Non significant at P ≤ 0. 001 using one way analysis of variance.

**Table 2:** **Effect of different concentrations of microcystins crude extract (25, 50, 100 and 200 μg/mL) of *Microcystis aeruginosa *** **in log phase on intra (IPS) and extra-cellular polysaccharides content (EPS) in μg/mL of *Oscillatoria angustissima***

**Microcystin crude extract concentrations (μg/mL)**								
**Polysaccharide**	**Days**	**Control**	**25**	**50**	**100**	**200**	**F value**	**P value**
	0	5±18	95±18^(ns)^	95±18^(ns)^	95±18^(ns)^	95±18^(ns)^		
	3	177±37	177±37^(ns)^	177±37^(ns)^	177±37^(ns)^	177±37^(ns)^		
IPS	6	355±38	387±33^(ns)^	425±29^***^	476±35^***^	530±34^***^	171.1	0.001
	9	403±34	440±21^(ns)^	512±20^***^	559±46^***^	712±38^***^		
	12	461±28	522±29^(ns)^	582±28^***^	728±30^***^	863±53^***^		
	15	550±45	598±23^(ns)^	758±62^***^	867±58^***^	1079±45^***^		
	0	156±24	156±24^(ns)^	156±24^(ns)^	156±24^(ns)^	156±24^(ns)^		
	3	285±31	285±31^(ns)^	285±31^(ns)^	285±31^(ns)^	285±31^(ns)^		
EPS	6	311±37	377±23^(ns)^	502±18^***^	525±28^***^	633±46^***^	1111.17	0.001
	9	631±28	830±69^***^	980±24^***^	1051±50^***^	1198±33^***^		
	12	1045±47	1155±32^***^	1327±31^***^	1571±35^***^	1939±44^***^		
	15	1630±34	1963±40^***^	2232±85^***^	2499±40^***^	3043±83^***^		

^***^ Highly significant at P ≤ 0.001 using one way analysis of variance (ANOVA).

^(ns)^ Non significant at P ≤ 0. 001 using one way analysis of variance.

The suggested model (concentrations, days and interception between them) indicated that the deviation in EPS and IPS content of *Anabaena* PCC.7120 were 99.88% and 98.8% for EPS and IPS, respectively (Table 3) and for *O*. *angustissima* were 99.81% and 98.8%, respectively (Table 4).

**Table 3:** **Analysis of variance of extra and intra-cellular polysaccharides of *Anabaena *** **PCC.7120 at different concentrations (25, 50, 100 and 200 μg/mL) of microcystins crude extract of *Microcystis aeruginosa***

**Extra-cellular polysaccharides**			
Treatment	F- value	P- value	Adjusted R^2^
Concentrations	733.68	0.001	99.88%
Days	5044.92	0.001	
Concentrations*days	132.10	0.001	
Intra-cellular polysaccharides			
Concentrations	222.96	0.001	99.8%
Days	1025.12	0.001	
Concentrations*days	26.62	0.001	

**Table 4:** **Analysis of variance of extra and intra-cellular polysaccharides of *Oscillatoria angustissima *** **at different concentrations (25, 50, 100 and 200 μg/mL) of microcystins crude extract of *Microcystis aeruginosa***

**Extra-cellular polysaccharides**			
Treatment	F-value	P-value	Adjusted R^2^
Concentrations	442.77	0.001	99.81%
Days	5767.13	0.001	
Concentrations* days	80.87	0.001	
Intra-cellular polysaccharides			
Concentrations	128.44	0.001	98.8%
Days	819.17	0.001	
Concentrations*days	18.49	0.001	

Results revealed that, EPS and IPS content of *Scendesmus obliquus* and *Chlorella vulgaris* showed increasing under different concentrations (25, 50, 100 and 200 μg/mL) of microcystins crude extract of *M*. *aeruginosa*. The increasing in EPS and IPS of *Sc*. *obliquus* were (41%, 75%, 112% and 182.5%) and (33.6%, 117.8%, 212.7% and 404%) over the control value for each concentration for EPS and IPS, respectively (Table 5). On the other hand, increasing in EPS and IPS of *C*. *vulgaris* amounted by (56%, 133%, 188% and 308.6%) and (25%, 60%, 309% and 478%), respectively over than control (Table 6).

**Table 5:** **Effect of different concentrations of microcystins crude extract (25, 50, 100 and 200 μg/mL) of *Microcystis aeruginosa *** **in log phase on intra (IPS) and extra-cellular polysaccharides content (EPS) in μg/mL of *Scendesmus obliquus***

**Microcystin crude extract concentrations (μg/mL)**								
**Polysaccharide**	**Days**	**Control**	**25**	**50**	**100**	**200**	**F value**	**P value**
	0	123±25	123±25^(ns)^	123±25^(ns)^	123±25^(ns)^	123±25^(ns)^		
	3	167±30	167±30^(ns)^	167±30^(ns)^	167±30^(ns)^	167±30^(ns)^		
IPS	6	177±35	213±25^(ns)^	258±33^**^	368±29^***^	446±42^***^	654.47	0.001
	9	230±37	251±35^(ns)^	376±30^***^	759±36^***^	920±40^***^		
	12	332±45	397±46^(ns)^	651±43^***^	1168±61^***^	1665±33^***^		
	15	488±49	652±47^***^	1014±41^***^	1526±45^***^	2460±60^***^		
	0	27±8	27±8^(ns)^	27±8^(ns)^	27±8^(ns)^	27±8^(ns)^		
	3	64.7±28	64.7±28^(ns)^	64.7±28^(ns)^	64.7±28^(ns)^	64.7±28^(ns)^		
EPS	6	169±29	198.7±11^(ns)^	220.6±39^(ns)^	286±14^***^	453±19^***^	185.94	0.001
	9	220±26	317.7±29^(ns)^	398±25^***^	416±20^***^	634±52^***^		
	12	279±25	434±32^***^	518±42^***^	636±47^***^	948±45^***^		
	15	378±26	534±36^***^	662±56^***^	803±53^***^	1068±114^***^		

^***^ Highly significant at P ≤ 0.001 using one way analysis of variance (ANOVA).

^(ns)^ Non significant at P ≤ 0. 001 using one way analysis of variance.

**Table 6:** **Effect of different concentrations of microcystins crude extract (25, 50, 100 and 200 μg/mL) of *Microcystis aeruginosa *** **in log phase on intra (IPS) and extra-cellular polysaccharides content (EPS) in μg/mL of *Chlorella vulgaris***

**Microcystin crude extract concentrations (μg/mL)**								
**Polysaccharide**	**Days**	**Control**	**25**	**50**	**100**	**200**	**F value**	**P value**
	0	72±20	72±20^(ns)^	72±20^(ns)^	72±20^(ns)^	72±20^(ns)^		
	3	122±20	122±20^(ns)^	122±20^(ns)^	122±20^(ns)^	122±20^(ns)^		
IPS	6	162±27	207±27^(ns)^	236±38^(ns)^	256±40^***^	325±42^***^	397.39	0.001
	9	227±30	260±29^(ns)^	352±36^***^	724±53^***^	984±78^***^		
	12	274±25	367±29^***^	435±37^***^	998±89^***^	1585±78^***^		
	15	369±30	463±38^***^	591±46^***^	1509±64^***^	2133±108^***^		
	0	54±17	54±17^(ns)^	54±17^(ns)^	54±17^(ns)^	54±17^(ns)^		
	3	116±18	116±18^(ns)^	116±18^(ns)^	116±18^(ns)^	116±18^(ns)^		
EPS	6	130±19	146±25^(ns)^	179±24^**^	322±24^***^	381±22^***^	302.29	0.001
	9	157±16	183±21^(ns)^	250±21^***^	408±24^***^	550±20^***^		
	12	203±15	230±21^(ns)^	324±33^***^	474±23^***^	781±20^***^		
	15	232±16	363±33^***^	541±21^***^	669±32^***^	948±51^***^		

^***^ Highly significant at P ≤ 0.001 using one way analysis of variance (ANOVA).

^(ns)^ Non significant at P ≤ 0. 001 using one way analysis of variance.

Analysis of variance revealed high significant increase in extra and intra-cellular polysaccharides of *Sc*. *obliquus* and *Chlorella vulgaris* at (P ≤ 0. 001) except at 25 μg/ml the increases in IPS of *Sc*. *obliquus* and EPS of *Chlorella vulgaris* (Tables 5 and 6, respectively) were insignificant at the days 6, 9 and 12, and the IPS of *C*.*vulgaris* was insignificant at days 6 and 9 as EPS of *Sc*. *obliquus*.

From the suggested statistical model (concentrations, days and interception between them), it could be observed that, the deviation for EPS and IPS content of *Sc*. *obliquus* was 98.89% and 99.68%, respectively (Table 7) and for *C*. *vulgaris* was 99.32% and 99.48%, respectively (Table 8).

**Table 7:** **Analysis of variance of extra and intra-cellular polysaccharides of *Scendesmus obliquus *** **at different concentrations of microcystins crude extract of *Microcystis aeruginosa***

**Extra**-**cellular polysaccharides**			
Treatment	F- value	P- value	Adjusted R^2^
Concentrations	221.27	0.0001	98.89%
Days	778.44	0.0001	
Concentrations * Days	30.10	0.0001	
Intra-cellular polysaccharides			
Concentrations	1117.14	0.0001	99.68%
Days	1991.37	0.0001	
Concentrations * Days	227.71	0.0001	

**Table 8:** **Analysis of variance of extra and intra-cellular polysaccharides of *Chlorella vulgaris *** **at different concentrations of microcystins crude extract of *Microcystis aeruginosa***

**Extra**-**cellular polysaccharides**			
Treatment	F- value	P- value	Adjusted R^2^
Concentrations	584.03	0.0001	99.32%
Days	964.36	0.0001	
Concentrations* Days	80.42	0.0001	
Intra-cellular polysaccharides			
Concentrations	746.23	0.0001	99.48%
Days	1061.04	0.0001	
Concentrations* Days	161.71	0.0001	

## Discussion

*Microcystis aeruginosa* blooms are hazardous to freshwater flora and fauna due to the production of toxins [25]. *M*. *aeruginosa* produces a wide range of toxic metabolites. The physiological and ecological role of these compounds remains largely unknown. Some studies have suggested that these compounds may have allelochemical roles, such as compounds that may inhibit competing sympatric macrophytes, algae and microbes [26,27]. The adverse effects of crude microcystins (MCs) could be due to the synergistic interactions among MCs variants or to the presence of unidentified toxic other than MCs in the crude extracts [28]. Leflaive and Ten-Hage [29] reported that cellular extracts containing toxins are often more active than purified toxin, they suggests that cellular extracts contain a mix of active toxin that may act synergistically.

The possible adaptive response of four unicellular algae (*Anabaena* PCC. 7120 and *O*. *angustissima* as blue green algae and *S*. *obliquus*; *C*. *vulgaris* as green algae) to *M*. *aeruginosa* producing toxin were studied. The results indicated that the crude microcystins extract induces the production of extra and intra-cellular polysaccharides of the tested algae and their amounts increased with increasing the microcystins crude extract concentration. The production of polysaccharide by green algal species under toxic stress indicated the involvement of this polysaccharide in protecting the algal cells against toxic species [13].

Inhibition in the growth of *Anabaena* PCC 7120; *Oscillatoria angustissima*; *Scendesmus obliquus* and *Chlorella vulgaris* at different microcystin crude extract concentrations was previously represented by [27]. Similar results were recorded by Abdel-Rahman [30] found that growth and the physiological activities, except amino acids biosynthesis, of both *Chlorococcum humicola* and *Chlorella vulgaris* were inhibited by crude extracts of the two cyanobacteria species *Microcystis* and *Nodularia*. Singh *et al*. [31] reported that the crude extract of *M*. *aeruginosa* provides toxicity to green algae (*Chlorella* sp. and *Scenedesmus* sp.) and cyanobacteria (*Anabaena* BT1 and *Nostoc muscorum*). As represented by [13] pure and crude microcystins significantly decreased the growth of *Scenedesmus quadricauda* and *Chlorella vulgaris*, and these results correlated with polysaccharide contents of toxin-treated cultures. This results was in accordance with our results which indicated that *S*. *obliquus* and *C*. *vulgaris* having a resistant system to crude toxin by the production of polysaccharide more than *Anabaena* PCC. 7120 and *O*. *angustissima*.

The increasing in polysaccharides contents in MCs treated cultures in all experimented algae indicated that these polysaccharides may be involved in certain defense mechanisms in response to toxin stress. Some studies have reported the protective role of polysaccharides against oxidative stress and their ability in scavenging ROS in plant cells [32]. The antioxidant mechanisms may be due to the supply of hydrogen by these polysaccharides which combines with radicals and forms more stable products to terminate radical chain reaction [33].

Microcystins have been shown to induce formation of reactive oxygen species (ROS) that might cause serious cellular damage such as peroxidation of lipid membranes, genotoxicity, or modulation of apoptosis [13]. During the present study the EPS and IPS were detected in the algal medium increased with increase of MCs concentration. It has been suggested that polysaccharides are produced inside the cells during oxidative stress to scavenge the free radicals and remove them from the cells to the medium [32]. However, a variety of other mechanisms such as extra-cellular detoxification, reduced uptake, efflux, sequestration by polysaccharides have been proposed to explain algal tolerance to oxidative stress [34].

Extrusion of EPS can serve as a boundary between cells and the surrounding environment; they could fulfill a protective role against desiccation, antibacterial agents or predation by protozoan [35]. Hence, the results of present study showed that some algae can develop a defense system by the production of polysaccharides to cope with oxidative stress induced by MCs, and this may explain why some plank tonic algae present in close proximity with toxic cyanobacteria or their toxins are not affected by these toxins at environmentally relevant concentrations (1–10 μL) [36].

## Conclusion

Here, we showed a physiological model of algal life sustainability under the stress of natural cyanobacterial toxins. The presented results showed the production of polysaccharides by tested algae as a response to the cyanobacterial toxins, microcystins. on the other hand, the release of these polysaccharides into the culture medium and most likely in the natural environment, is of ecologically importance because they may increase the polysaccharide contents of the water column and the growth of heterotrophic bacteria, and complex with heavy metals to reduce their toxicity to aquatic organisms. Therefore, in future studies involving *in vitro* cultures more attention should be paid to the role of algal exudates, in order to improve the significance of the results with the aim of using them as models of the real environment.

## Competing interests

The authors declare that they have no competing interests.

## Authors’ contributions

This work is part of the Master thesis of RE where MME, supervised the thesis, suggested the problem and wrote the paper and MME participated in writing the paper and HMK helped in experiments and read the paper. All authors read and approved the final manuscript.
